# Supplementary material for: Topological Fermi-arc surface state covered by floating electrons on a two-dimensional electride
Source: Nat Commun. 2024 Jul 4;15:5615. doi: 10.1038/s41467-024-49841-6 (PMC11224405; doi:10.1038/s41467-024-49841-6)
Supplement: Supplementary file 1 — Supplementary Information [file 41467_2024_49841_MOESM1_ESM.pdf]

Supplementary Information for Topological Fermi-arc surface state covered by floating electrons in a two-dimensional electride

Chan-young Lim[1,†,§], Min-Seok Kim[2,†], Dong Cheol Lim[3,4,†], Sunghun Kim[5,†], Yeonghoon Lee[6], Jaehoon Cha[1], Gyubin Lee[1], Sang Yong Song[2], Dinesh Thapa[7], Jonathan D. Denlinger[8], Seong-Gon Kim[9*], Sung Wng Kim[3,4,*], Jungpil Seo[2,*] and Yeongkwan Kim[1,*]

[1]Department of Physics, Korea Advanced Institute of Science and Technology, Daejeon 34141, Korea.

[2]Department of Physics and Chemistry, Daegu Gyeongbuk Institute of Science and Technology, Daegu 42988, Korea.

[3]Department of Energy Science, Sungkyunkwan University, Suwon 16419, Korea.

[4]Center for Electride Materials, Sungkyunkwan University, Suwon 16419, Korea.

[5]Department of Physics, Ajou University, Suwon 16499, Korea

[6]Quantum Spin Team, Korea Research Institute of Standards and Science, Daejeon 34113, Korea.

[7]Department of Chemistry and Biochemistry, North Dakota State University, Fargo, North Dakota 58108, USA.

[8]Advanced Light Source, Lawrence Berkeley National Laboratory, Berkeley, California 94720, USA.

[9]Department of Physics & Astronomy and Center for Computational Sciences, Mississippi State University, Mississippi States, Mississippi 39792, USA.

†These authors contributed equally to this work.

§Current affiliation: Donostia International Physics Center (DIPC), San Sebastián/Donostia 20018, Spain

*E-mail: sk162@msstate.edu, kimsungwng@skku.edu, jseo@dgist.ac.kr, yeongkwan@kaist.ac.kr

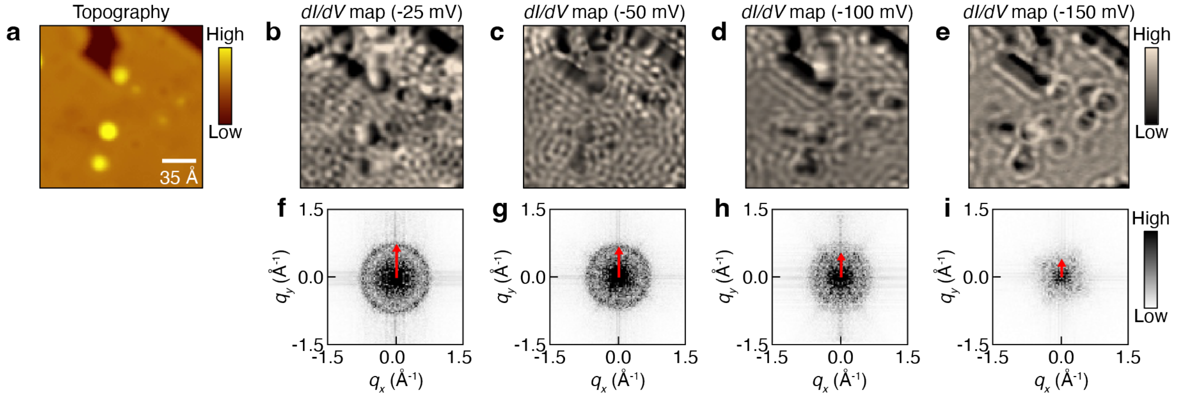

Supplementary Figure 1 | QPI measurements on floating electrons of $[Gd_2C]^{2+}\cdot 2e^-$. a, Topographic image of floating electrons of $[Gd_2C]^{2+}\cdot 2e^-$. **b-e**, *dI/dV* maps measured at -25 mV (**b**), -50 mV (**c**), -100 mV (**d**) and -150 mV (**e**). I_t = 100 pA and V_{mod} = 10 mV. **f-i**, Fourier transformed images of **b-e**, respectively.

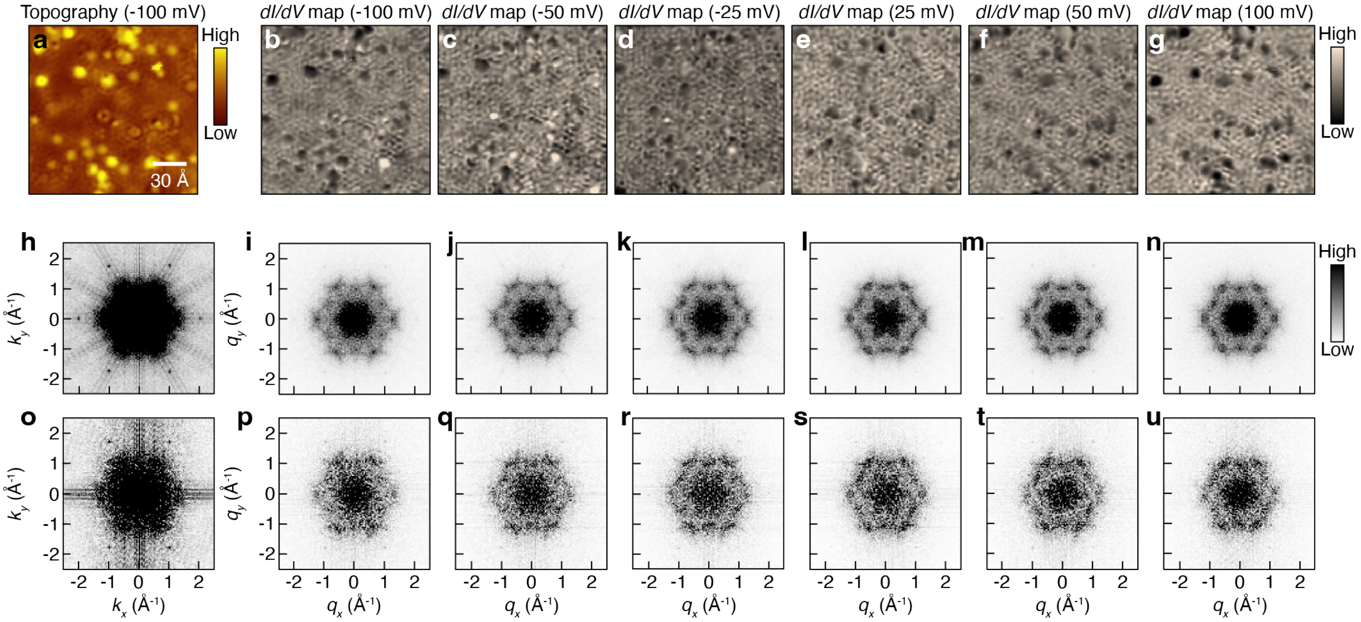

Supplementary Figure 2 | QPI measurements on Fermi-arc states of $[Gd_2C]^{2+}\cdot 2e^-$. **a**, Topographic image of $[Gd_2C]^{2+}$ surface. **b-g**, *dI/dV* maps measured at -100 mV (**b**), -50 mV (**c**), -25 mV (**d**), 25 mV (**e**), 50 mV (**f**) and 100 mV (**g**). I_t = 100 pA and V_{mod} = 10 mV. **h**, Fourier transformed image of **a**. The image is symmetrized three-fold to enhance the signal-to-noise ratio. **i-n**, Fourier transformed images of **b-g**, respectively. The images are symmetrized three-fold. **o-u**, Raw data of **h-n** before the symmetrisation.

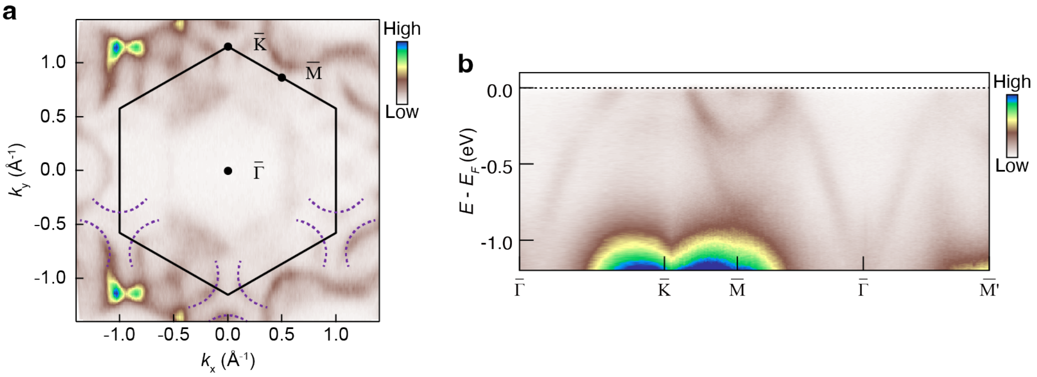

Supplementary Figure 3 | ARPES measured electronic structure of $[Gd_2C]^{2+}\cdot 2e^-$ without the floating electrons. a, Fermi surface of $[Gd_2C]^{2+}\cdot 2e^-$ measured with 90 eV photon energy, after the heating cycle. The circular Fermi surface of the floating electron at the zone center is missing, compared to Fig 1e of the main text. The 1st BZ boundaries are depicted with black solid lines, and the purple dashed lines near $\overline{K}$ points indicate Fermi arcs. **b,** The dispersions along the multiple high symmetry lines. While overall dispersion is maintained, the dispersion is slightly shifted compared to the pristine case, indicating there is a Fermi level shift due to the removal of floating electrons.

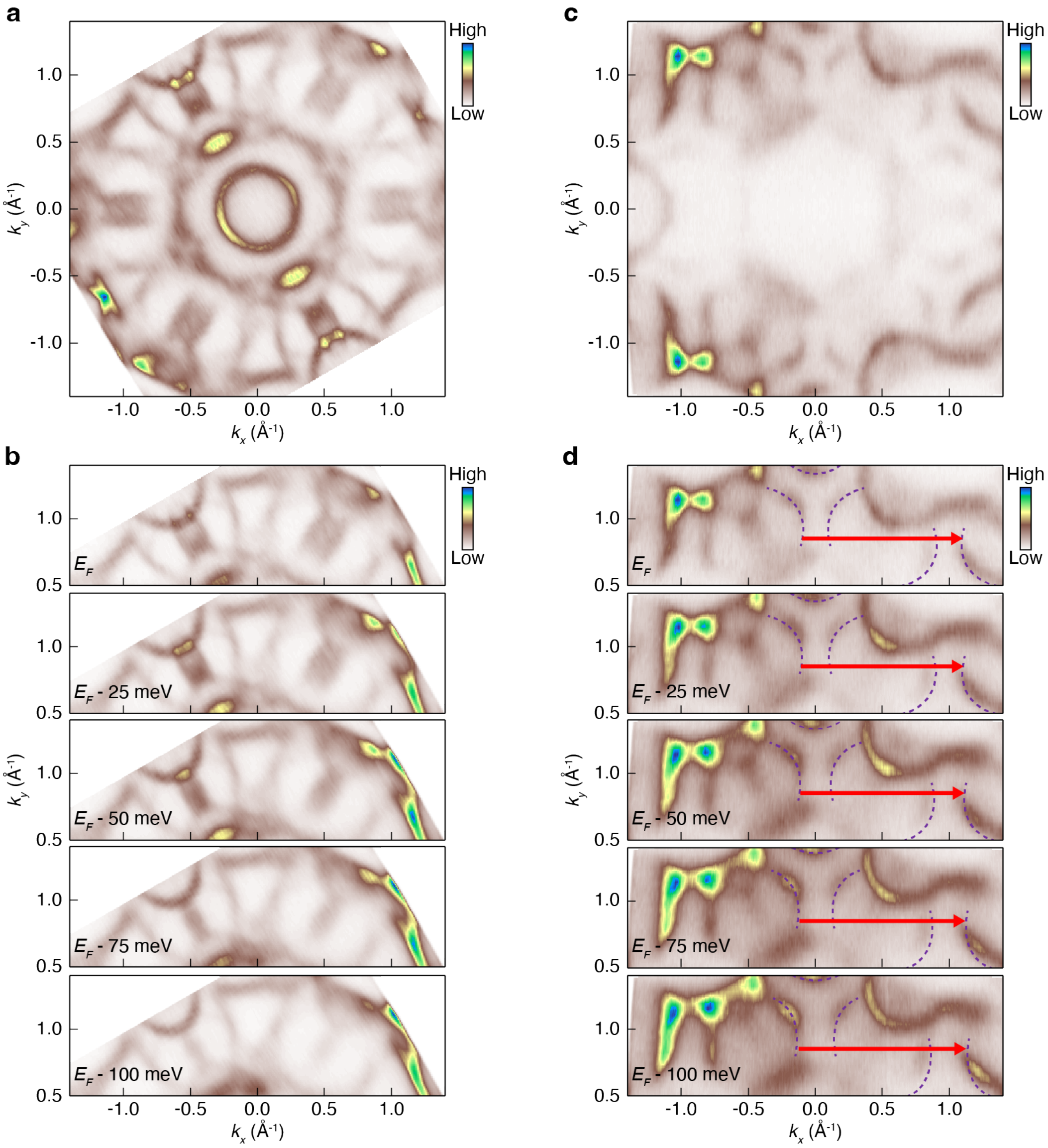

Supplementary Figure 4 | Comparison between the Fermi surfaces of $[Gd_2C]^{2+}\cdot 2e^-$ with and without the floating electrons. a, Fermi surface of $[Gd_2C]^{2+}\cdot 2e^-$ measured in the presence of the floating electrons. **b,** Constant energy contours of $[Gd_2C]^{2+}\cdot 2e^-$ near $\overline{\mathrm{K}}$ point with the floating electrons at binding energies ranging from the Fermi level to 100 meV. **c,d**, Same set of data from the floating electron removed sample. Purple dashed lines serve as guides for the Fermi arcs, and red arrows correspond to the $\mathbf{q}_2$ vector from the main text, demonstrating almost identical nesting conditions within the binding energy range of interest.
